# Supplementary material for: Preferent Diaphragmatic Involvement in TK2 Deficiency: An Autopsy Case Study
Source: Int J Mol Sci. 2021 May 25;22(11):5598. doi: 10.3390/ijms22115598 (PMC8199166; doi:10.3390/ijms22115598)
Supplement: Supplementary file 1 [file ijms-22-05598-s001.zip › Figure S1 R1.pdf]

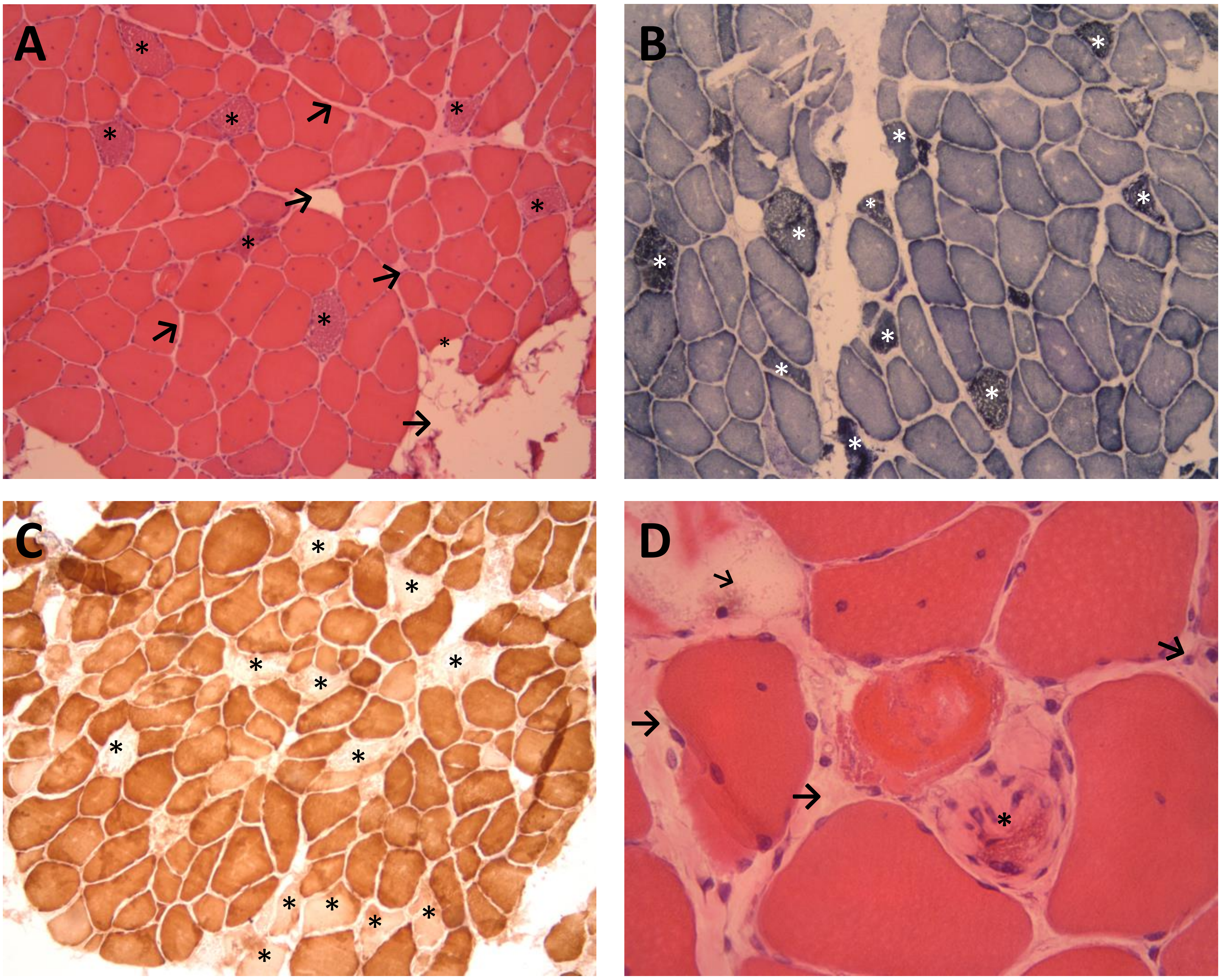

Figure S1. **Histological analysis of *post mortem* quadriceps.** Representative images of muscle sections showing numerous ragged-red fibers (A); hyper-reactive succinate dehydrogenase (SDH) fibers (B); cytochrome-c oxidase (COX) negative fibers (C); and necrotic fibers with myophagocytosis (D). *Altered fibers. Arrows, endomysial fibrosis and adipose tissue replacement. A-C 10x, and D 40x.
