# Supplementary material for: Preferent Diaphragmatic Involvement in TK2 Deficiency: An Autopsy Case Study
Source: Int J Mol Sci. 2021 May 25;22(11):5598. doi: 10.3390/ijms22115598 (PMC8199166; doi:10.3390/ijms22115598)
Supplement: Supplementary file 1 [file ijms-22-05598-s001.zip › Figure S2 R1.pdf]

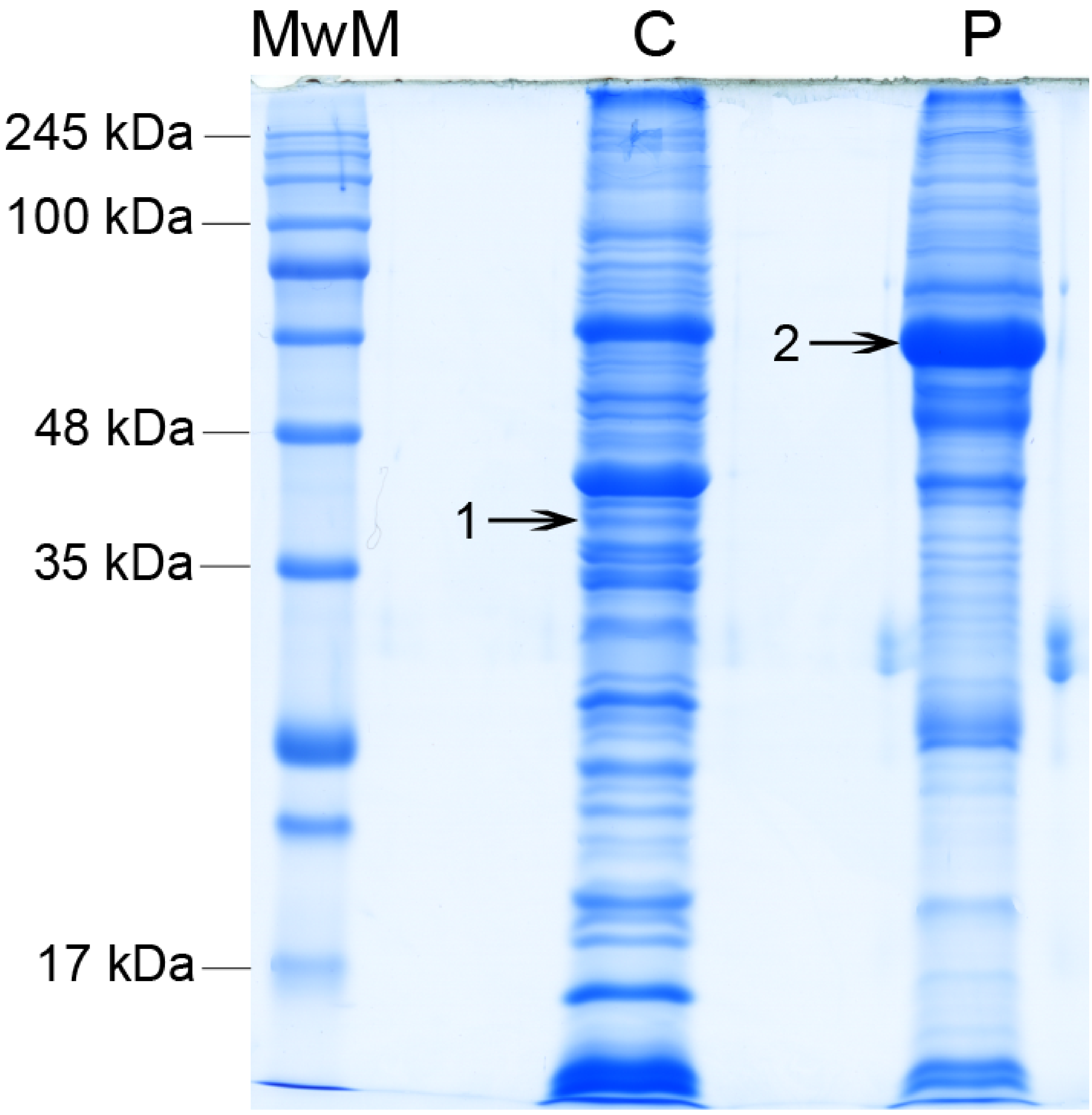

Figure S2. Protein profile in diaphragm. SDS-PAGE gel stained with Coomassie Blue showing the protein profile in diaphragm homogenates of a control (C) and the patient (P). Arrows:1, band present in control diaphragm and absent in the patient; 2 Major band enriched in the patient. MwM, molecular weight markers.
