# Supplementary material for: Preferent Diaphragmatic Involvement in TK2 Deficiency: An Autopsy Case Study
Source: Int J Mol Sci. 2021 May 25;22(11):5598. doi: 10.3390/ijms22115598 (PMC8199166; doi:10.3390/ijms22115598)
Supplement: Supplementary file 1 [file ijms-22-05598-s001.zip › Figure S3.pdf]

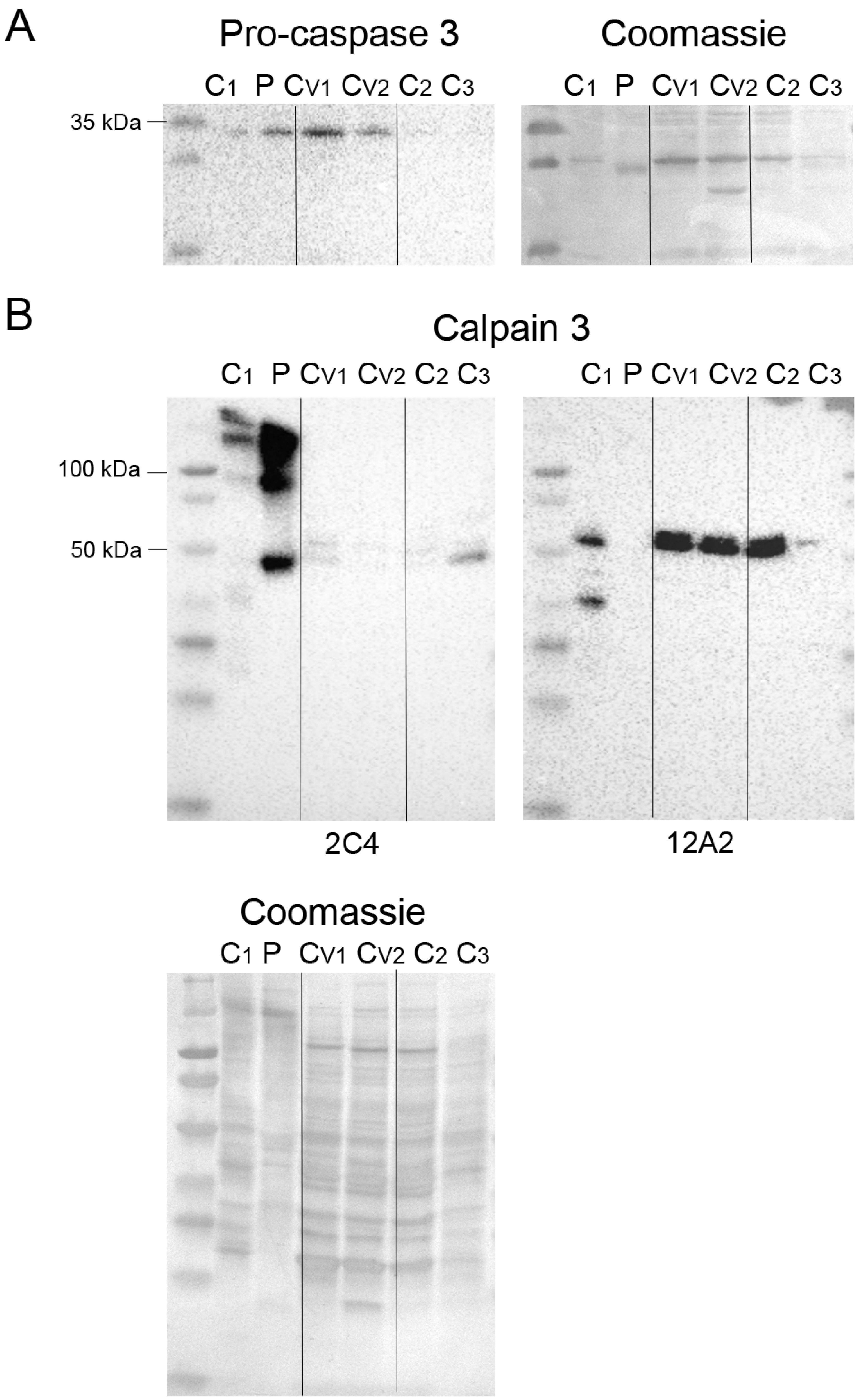

Figure S3. Proteases in diaphragm. Representative western blotting of pro-caspase 3 (A) and calpain 3 (B) using antibodies 2C4 and 12A2 in diaphragm homogenates of non-ventilated controls (C, n=3), ventilated controls (C_v, n=2), and the patient (P). Coomassie straining of the membranes is shown as total protein loading control.
