# Supplementary material for: Preferent Diaphragmatic Involvement in TK2 Deficiency: An Autopsy Case Study
Source: Int J Mol Sci. 2021 May 25;22(11):5598. doi: 10.3390/ijms22115598 (PMC8199166; doi:10.3390/ijms22115598)
Supplement: Supplementary file 1 [file ijms-22-05598-s001.zip › Figure S4 R1.pdf]

**Figure S4**. Original gels and membranes

**Figure 1**. LR_PCR in all tissues

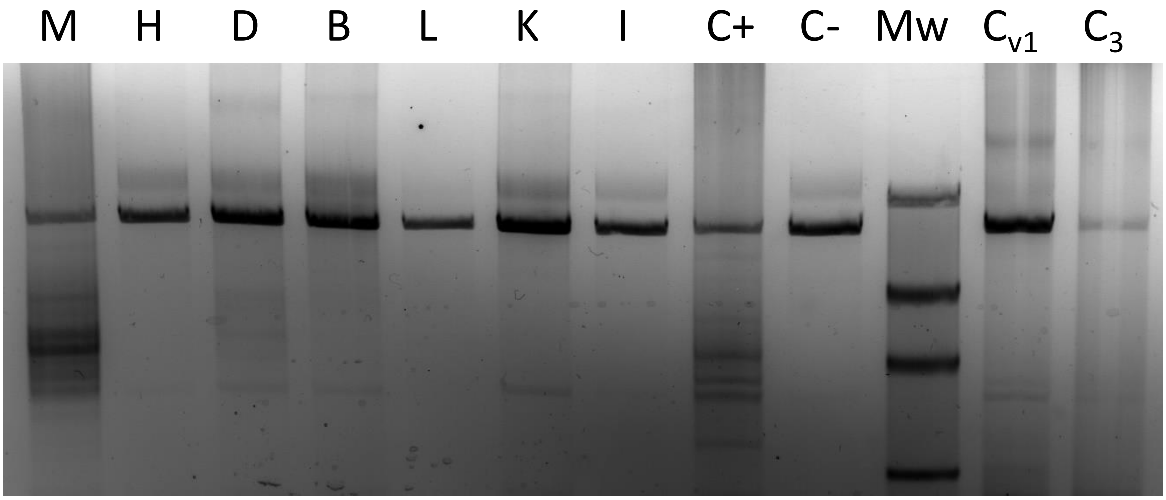

**Figure 2**. Western blot of OXPHOS proteins

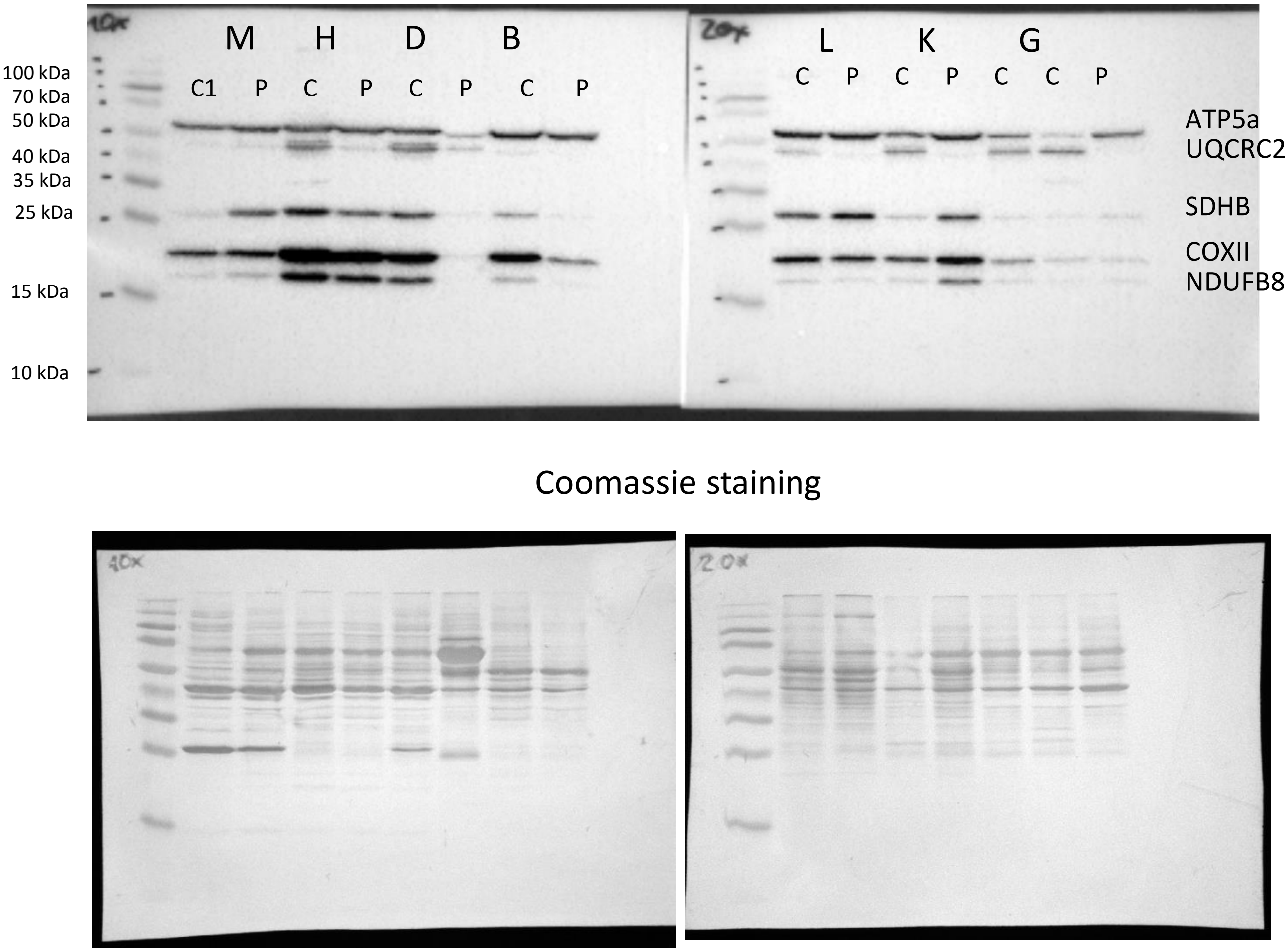

**Figure 2 and Figure 4**. Western blot of OXPHOS proteins (diaphragms and muscles)

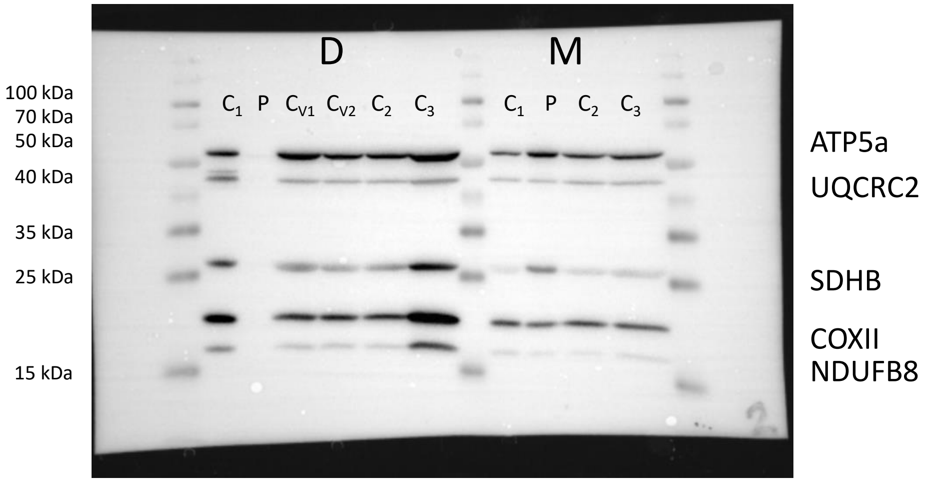


Coomassie staining

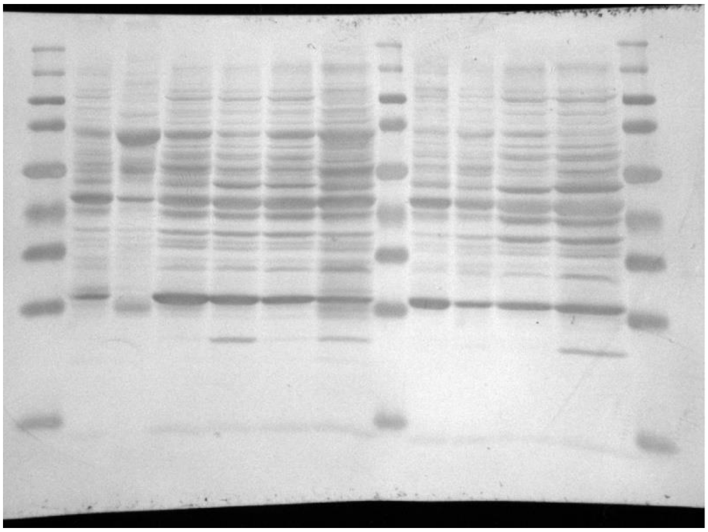

**Figure 3**. Constitutive proteins.

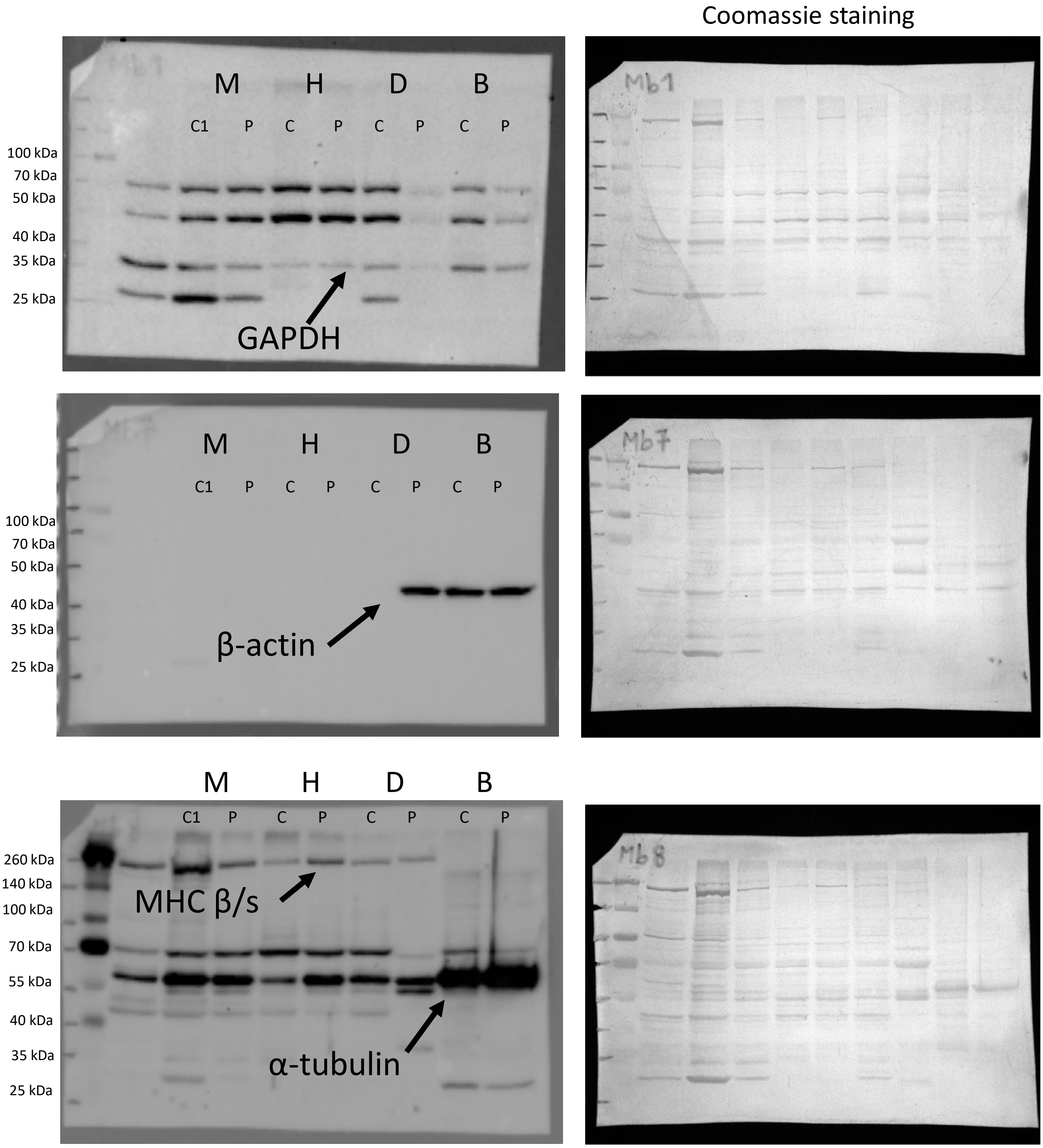


In these membranes and those in Figure 7 an additional sample unrelated to TK2 deficiency which is not included in the manuscript was loaded on the left side of the gels.

**Figure S2**. Protein profile in diaphragm

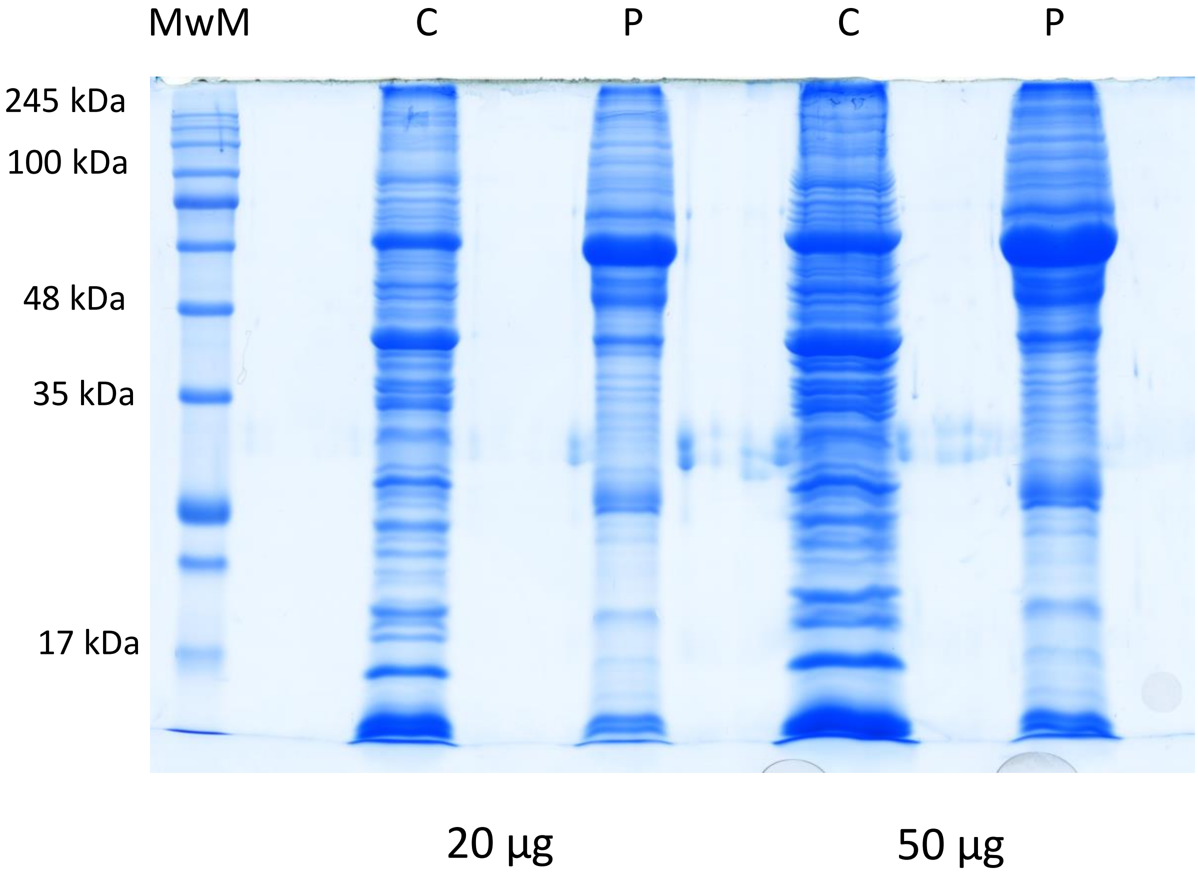

**Figure 5**. Actin isoforms and TK2 in diaphragm.

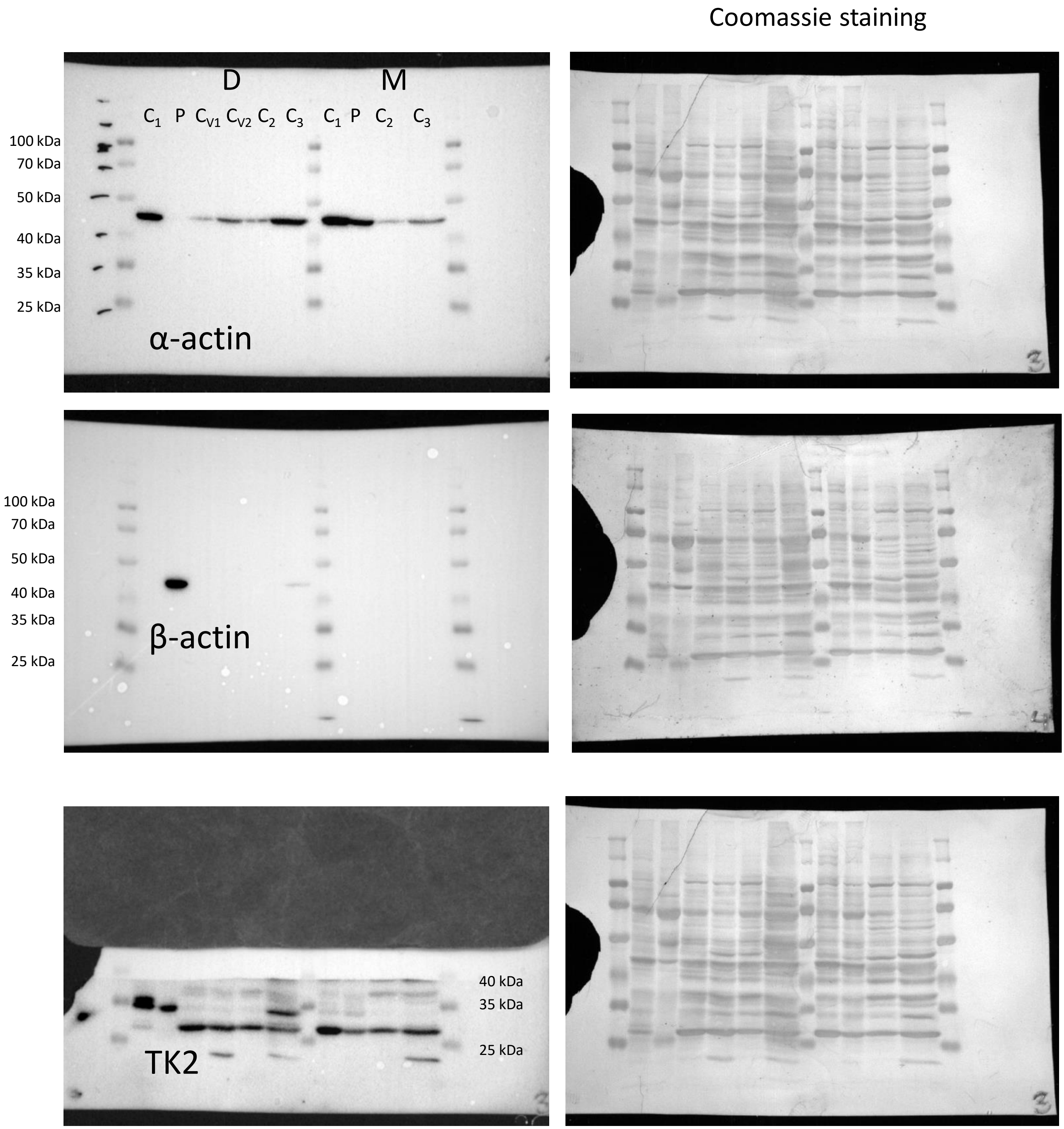


TK2 image obtained covering the upper part of the membrane because very intense upper bands interfered with TK2 signal.

**Figure 6**. Antioxidant enzymes.

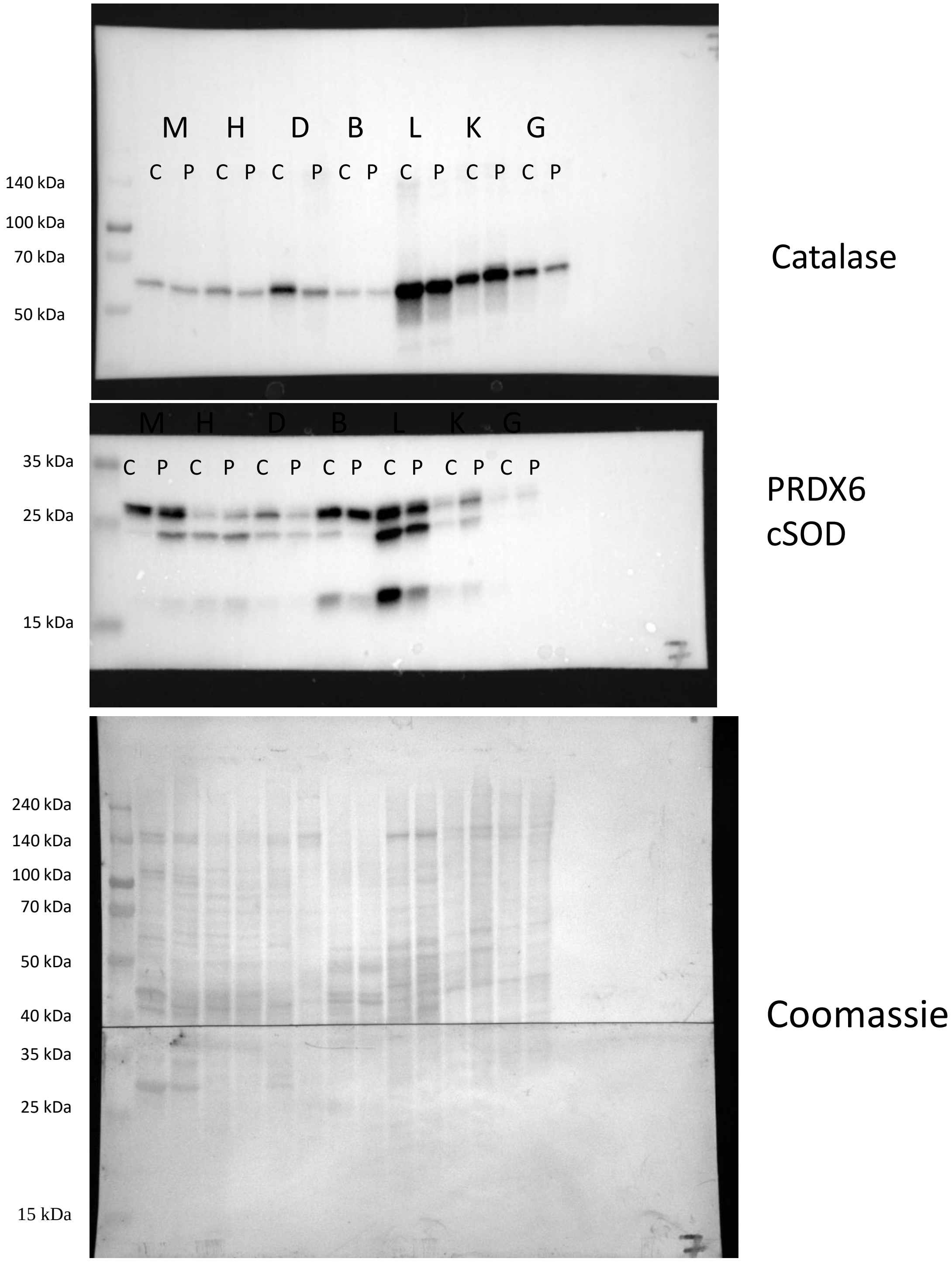

**Figure 7.** Autophagy and ubiquitin-proteasome system markers.

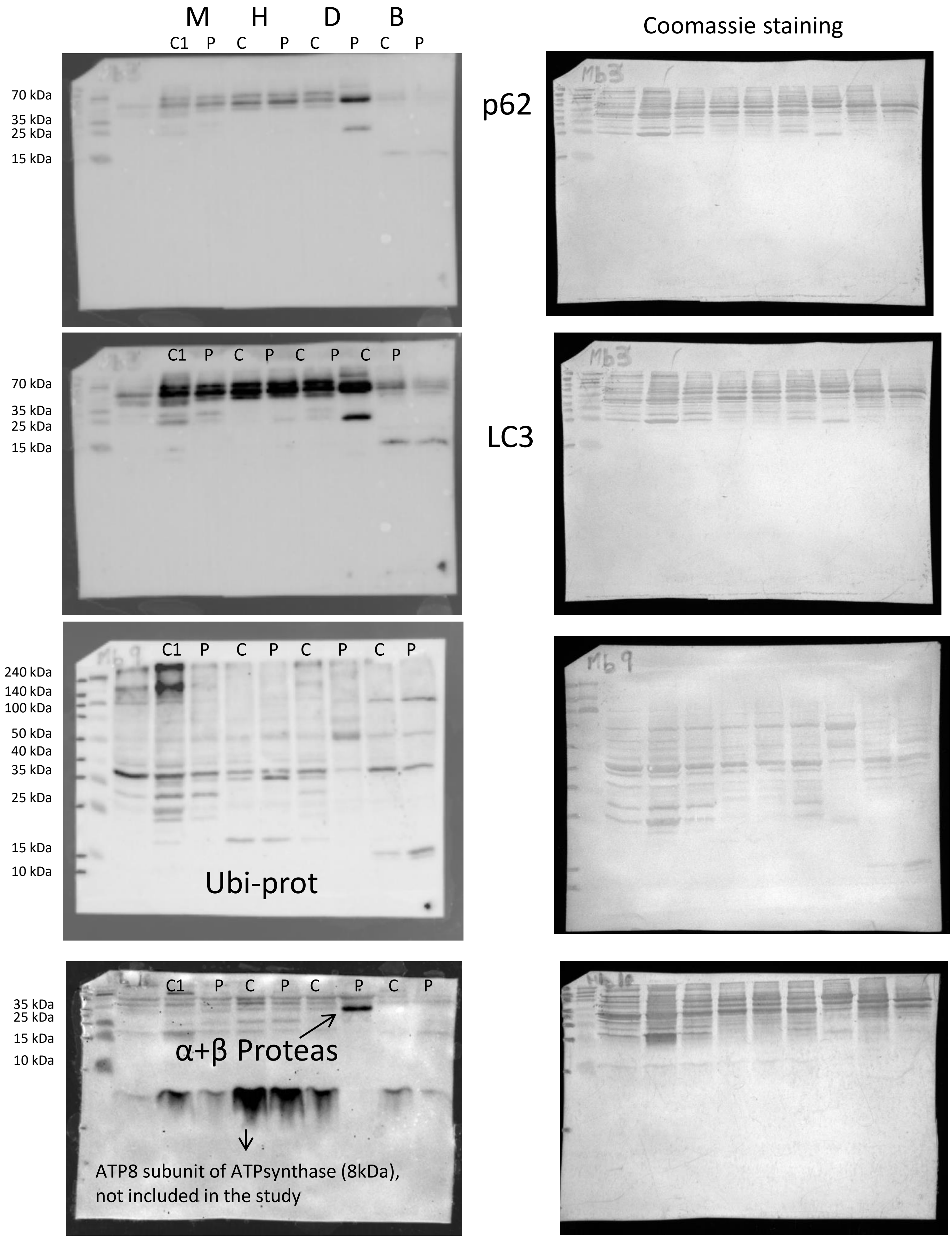

**Figure 8 and Figure S3.** Whole membranes with molecular weigh markers are shown in the manuscript figures.
