# Supplementary material for: Preferent Diaphragmatic Involvement in TK2 Deficiency: An Autopsy Case Study
Source: Int J Mol Sci. 2021 May 25;22(11):5598. doi: 10.3390/ijms22115598 (PMC8199166; doi:10.3390/ijms22115598)
Supplement: Supplementary file 1 [file ijms-22-05598-s001.zip › Table S1 R1.pdf]

Table S1.

Primary antibody	Reference	Dilution (for WB)
α-Actin	Gene Tex GTX101362	1:2.000
α+β Proteas	Abcam ab22673	1:1.000
β-Actin	Sigma A5316	1:5.000
Calpain 3 (2C4)	GeneTex GTX01971	1:50
Calpain 3 (12A2)	GeneTex GTX01970	1:100
Catalase	Sigma C0979	1:1.000
cSOD	Enzo ADI-SOD-100	1:1.000
GAPDH	SIGMA G9545	1:10.000
LC3	Sigma L7543	1:1.000
MHC-β/s	Aviva Systems ARP41380_P050	1:1.000
OXPHOS cocktail	Abcam ab110411	1:500
p62	Abcam ab109012	1:10.000
PRDX6	GeneTex GTX115262	1:1.000
Pro-caspase 3	Cell Signalling #9662	1:1.000
TK1	Abcam ab76495	1:5.000
TK2	Sigma SAB1300098	1:500
α-Tubulin	BioRAD VPA00655	1:2.000
Ubi-prot	Abcam ab7780	1:1.000

WB, western blot.
